# The HTLV-1 Tax protein inhibits nonsense-mediated mRNA decay by interacting with INT6/EIF3E and UPF1

**DOI:** 10.1186/1742-4690-8-S1-A139

**Published:** 2011-06-06

**Authors:** Vincent Mocquet, Julia Neusiedler, Francesca Rende, Jean-Michel Terme, Christelle Morris, Jürgen Wittman, Madeleine Duc Dodon, Vincenzo Ciminale, Pierre Jalinot

**Affiliations:** 1 Laboratoire de Biologie Moléculaire de la Cellule, UMR 5239, Ecole Normale Supérieure de Lyon, Lyon, 69364, cedex 07, France; 2 Department of Oncology and Surgical Sciences and Istituto Oncologico Veneto-Istituto di Ricovero e Cura a Carattere Scientifico (IRCCS), Padova, Italy; 3 Division of Molecular Immunology, Department of Internal Medicine III, Nikolaus-Fiebiger- Center, University of Erlangen-Nürnberg, Erlangen, D-91054, Germany; 4 Virologie Humaine, Unité 758, Institut National de la Santé et de la Recherche Médicale, Ecole Normale Supérieure de Lyon, Institut Fédératif de Recherche 128 Biosciences Lyon Gerland, Lyon, cedex 07, France

## 

The Nonsense Mediated mRNA Decay (NMD) regulates the expression of many genes such as GADD45a and lead to the degradation of mRNA exhibiting a premature STOP codon (PTC). The cellular protein INT6 has been identified by our team as a major actor of this pathway. We also demonstrated that INT6 interacts with Tax, the transcriptional activator of HTLV-1 that plays a major role in the cellular transformation associated to HTLV. As a consequence, a blockade of the NMD pathway by Tax through INT6 binding could alter the genetic expression profil of an infected cell and play a role in the emergence of a transformed clone.

We first showed that Tax increases the half-life of PTC containing reporter genes as well as endogenous targets of the NMD such as gadd45a. Then we decrypted by immunoprecipitation experiments, a network of interactions between the viral protein, INT6 and the UPFs. These results, combined with confocal microscopy observations, suggest that Tax (1) sequesters INT6 out of reach from UPF1/2 and (2) interacts with phospho-UPF1, inhibiting its dephosphorylation which is indispensable during NMD. Interestingly, Tax causes a strong increase in the size and number of P.bodies, where UPF1 accumulates. Finally, RNA immunoprecipitations demonstrated that Tax is associated with the mRNA targeted by the NMD pathway.

Collectively these data show that in addition to its role in transcriptional activation, Tax can also interfere at the posttranscriptional level with the regulation of mRNA degradation through the NMD pathway.

